# High impact nutrition and dietetics journals’ use of publication procedures to increase research transparency

**DOI:** 10.1186/s41073-020-00098-9

**Published:** 2020-08-31

**Authors:** Dennis M. Gorman, Alva O. Ferdinand

**Affiliations:** 1 Department of Epidemiology & Biostatistics, School of Public Health, Texas A&M University, College Station, TX USA; 2 Department of Health Policy & Management, School of Public Health, Texas A&M University, College Station, TX USA

**Keywords:** Nutrition and dietetics research, Research integrity, Conflict of interest, Study registration, Study guidelines, Data transparency

## Abstract

**Background:**

The rigor and integrity of the published research in nutrition studies has come into serious question in recent years. Concerns focus on the use of flexible data analysis practices and selective reporting and the failure of peer review journals to identify and correct these practices. In response, it has been proposed that journals employ editorial procedures designed to improve the transparency of published research.

**Objective:**

The present study examines the adoption of editorial procedures designed to improve the reporting of empirical studies in the field of nutrition and dietetics research.

**Design:**

The instructions for authors of 43 journals included in Quartiles 1 and 2 of the Clarivate Analytics’ 2018 Journal Citation Report category *Nutrition and Dietetics* were reviewed. For journals that published original research, conflict of interest disclosure, recommendation of reporting guidelines, registration of clinical trials, registration of other types of studies, encouraging data sharing, and use of the Registered Reports were assessed*.* For journals that only published reviews, all of the procedures except clinical trial registration were assessed.

**Results:**

Thirty-three journals published original research and 10 published only reviews. Conflict of interest disclosure was required by all 33 original research journals. Use of guidelines, trial registration and encouragement of data sharing were mentioned by 30, 27 and 25 journals, respectively. Registration of other studies was required by eight and none offered Registered Reports as a publication option at the time of the review. All 10 review journals required conflict of interest disclosure, four recommended data sharing and three the use of guidelines. None mentioned the other two procedures.

**Conclusions:**

While nutrition journals have adopted a number of procedures designed to improve the reporting of research findings, their limited effects likely result from the mechanisms through which they influence analytic flexibility and selective reporting and the extent to which they are properly implemented and enforced by journals.

## Introduction

The rigor and integrity of the published research literature in the field of nutrition studies has come into serious question in recent years. One reason for this is the publicity surrounding the retraction of more than a dozen papers produced by researchers at a major nutrition laboratory at one of the top universities in the United States [1, 2]. Such a large-scale retraction of papers casts doubts on the peer review process of the journals that published this research, and is especially concerning as many of these were high impact journals with presumably stringent peer review procedures and standards.

In addition, some nutrition researchers have begun to question the quality of published research both within the discipline as a whole and within specific areas of specialization. A report from an American Society for Nutrition advisory committee highlighted the threats to research integrity that arise from competing interests among researchers, specifically the type of selective statistical analyses that are conducted and reported and the conclusions investigators draw from these (e.g., withholding unfavorable findings from publication) [3]. In a more detailed examination of such issues in the field of childhood obesity interventions, Brown et al. identified ten statistical and methodological errors frequently found in the published literature that were associated with exaggerated claims about program effectiveness in changing diet, increasing exercise and weight reduction [4]. These included reporting results only for secondary outcome variables in the light of null findings for primary outcomes, reporting only subgroup analyses in the light of no main effects, data dredging for spurious statistically significant results, using one-tailed tests of statistical significance, and claiming that null results were nonetheless “clinically significant”. Bero and colleagues also identified bias in outcome reporting in a series of systematic reviews focused on nutrition research funded by the food industry [5–7]. They found the reporting of results in such studies tended to be skewed in favor of the sponsoring industry, and that authors’ financial conflicts of interest were frequently undisclosed in journal publications. Such reporting bias and lack of transparency is especially problematic as it can lead to the development of inappropriate dietary guidelines [8].

In the field of nutritional epidemiology, Ioannidis contends that “good scientific principles” are lacking, with investigators being free to report selective results from multiple analyses they have conducted, very few of which will have been prespecified [9]. In a study of published case-control and cohort studies that examined the association between 40 randomly selected ingredients from recipes and cancer, Schoenfeld and Ioannidis found that the vast majority reported either an increased or decreased risk based on very weak statistical evidence [10]. They attributed this to publication bias and selective reporting of positive results in publications.

The concerns raised regarding the quality and integrity of research published in the field of nutrition research reflect those that have arisen within many academic disciplines over the past decade, and that have been described in terms of a “credibility crisis” [11, 12]. The response to this crisis has been, in part, to call for increased rigor and transparency in the editorial and peer review process used by academic journals [13–15]. This has involved requiring full and detailed disclosure of conflicts of interest, use of guidelines such as the Consolidated Standards for Reporting Trials (CONSORT) [16] and Preferred Reporting Items for Systematic Reviews and Meta-Analyses (PRISMA) [17] when writing-up manuscripts for publication, preregistration of hypotheses, study designs and measures, and sharing of data and code as a condition of publication. Those concerned with the quality and integrity of nutrition research have also suggested that such procedures be introduced by academic journals within the discipline [3, 4].

Six studies have examined journal adoption of three editorial procedures designed to improve the quality of published research in the fields of pediatric research [18, 19], surgery [20], emergency medicine [21], orthopedic and general medicine [22], and addiction [23]. Clinical trial registration and recommendation of the CONSORT guidelines were assessed in all six studies, with adoption of the procedure ranging from 23 to 86% for the former and 20 to 81% for the latter [18–22]. Other reporting guidelines, such as PRISMA, were less likely to be recommended in the instructions for authors of these journals. Conflict of interest disclosure was examined in three studies and required by 61% of online and 78% of print pediatrics journals [18, 19], and 97% of addiction journals [23].

To our knowledge, no existing study has assessed the adoption of editorial procedures designed to improve the reporting of empirical studies in the field of nutrition research. The current study is intended to fill this gap in existing knowledge. It is an exploratory and descriptive study in which no hypotheses were prespecified or tested.

## Materials and methods

In line with previous studies of journal editorial procedures [18, 20–23], Clarivate Analytics Journal Citation Report (JCR) was used to identify high impact nutrition and dietetics journals. Specifically, 21 journals ranked in Quartile 1 (Q1) and 22 in Quartile 2 (Q2) of the 2018 JCR category *Nutrition and Dietetics* were selected [24]. The impact factor of each of these 43 journals was also obtained from the JCR webpage. Q1 journals are those with impact factors greater than 75% of journals within that particular JCR category, while Q2 journals have impact factors greater than 50% of journals within the category. The 43 Q1 and Q2 journals were selected so that their instructions for authors could be identified and reviewed in a timely manner (i.e., before they became outdated and were replaced online) and this number was considered to be reasonable in terms of representing the high impact journals within the field of nutrition and dietetics research. However, recognizing the limitations of journal rankings based on impact factor [25], the Scimago Journal Ranking (SJR) h-index of each of the 43 journals was also identified. This expresses the number of articles (*h*) published by the journal that have received at least h citations [26].

The instructions for authors of each of the 43 journals was located on the journal webpage on the Internet. In addition, if a journal’s instructions for authors cited additional documents that prospective authors were required to consult (e.g., publisher ethics guidelines), these too were located. Instructions for authors and related documents that were available as a Portable Document Format (PDF) were downloaded. If the author instructions and related documents on webpages were unavailable as a PDF, they were copied and pasted into a Word document. All the material reviewed was downloaded between April 3 and October 14, 2019. The downloaded instructions for authors of each journal, along with any relevant additional materials cited in these, were reviewed separately by each of the two authors and mention of each publication procedure was recorded. The two reviewers met to discuss their ratings and any discrepancies were resolved. Specifically, where there was disagreement about the presence or absence of requirements and recommendations in a particular set of instructions for authors, they re-reviewed these during the meeting and came to agreement as to whether the procedure was or was not described in the documents. The Center for Open Science list of journals that have adopted the Registered Reports publication format was also reviewed for the inclusion of any of the 43 nutrition and dietetics journals on December 19, 2019 [27].

For journals that published both original research and systematic reviews the following six procedures were assessed: (i) conflict of interest disclosure, (ii) recommendation of specific reporting guidelines when writing-up the results of studies for publication (e.g., CONSORT, PRISMA) or reference to the EQUATOR Network or FAIRshare repositories of guidelines [28, 29], (iii) registration of clinical trials in a registry such as Clinical Trials.gov [30] (iv) registration of other types of studies in a registry such as PROSPERO (for systematic reviews) [31], (v) encouraging or requiring data sharing, and (vi) use of the Registered Reports publishing format [27]*.* For journals that exclusively published systematic reviews and meta-analyses, five of the six procedures were considered relevant, the exception being clinical trial registration. In addition, in the case of two of the remaining five, it was anticipated that fewer guidelines would be relevant to journals that only publish reviews (e.g., PRISMA and Meta-analysis of Observational Studies in Epidemiology (MOOSE) [32]) and that just one registry (PROSPERO) would likely be utilized. These procedures were selected based on those used in previous studies of journal editorial practices [18–23], as well as those described in the broader literature on measures to reduce publication and reporting bias and promote research transparency [33–35].

The analyses presented are descriptive data pertaining to the number of publication procedures mentioned in journal instructions to authors.

## Results

Table 1 shows the JCR impact factor, SJR h-index and publisher of the 43 journals that appeared in the top two quartiles (Q1 and Q2) of the 2018 JCR *Nutrition and Dietetics* category. Ten of the journals were judged to be exclusively focused on review articles from the aims described in their instructions for authors, and these were reviewed separately from the 33 journals that focused on original research (in addition to reviews).

**Table 1:** 2018 JCR Q1 and Q2 Nutrition and Dietetics Journals Arranged by JCR and Scimago Impact Factors and with Publisher^a^

**Rank**	**Full Journal Title**	**Publisher**	**JCR Impact Factor**	**Scimago Impact Factor**^**b**^
*1*	*Progress in Lipid Research*^*c*^	Elsevier Ltd.	12.540	132
*2*	*Annual Review of Nutrition*^*c*^	Annual Reviews Inc.	8.422	142
*3*	*Advances in Nutrition*^*c*^	Oxford Academic	7.240	69
*4*	*Critical Reviews in Food Science & Nutrition*^*c*^	Taylor & Francis	6.704	135
*5*	*American Journal of Clinical Nutrition*	American Society for Clinical Nutrition	6.568	307
*6*	*Clinical Nutrition*	Elsevier BV	6.402	121
*7*	*International Journal of Behavioral Nutrition & Physical Activity*	BioMed Central	6.037	95
*8*	*Nutrition Reviews*^*c*^	Oxford Academic	5.779	131
*9*	*Nutrition Research Reviews*^*c*^	Cambridge University Press	5.595	71
*10*	*Food Chemistry*	Elsevier BV	5.399	221
*11*	*Proceedings of the Nutrition Society*^*c*^	Cambridge University Press	5.017	113
*12*	*International Journal of Obesity*	Nature Publishing Group	4.514	204
*13*	*Journal of Nutritional Biochemistry*	Elsevier BV	4.490	118
*14*	*European Journal of Nutrition*	Dr. Dietrich Steinkopff Verlag	4.449	85
*15*	*Journal of Nutrition*	American Society for Nutritional Sciences	4.416	240
*16*	*Nutrients*	Multidisciplinary Digital Publishing Institute	4.171	75
*17*	*Journal of the Academy of Nutrition & Dietetics*	Elsevier USA	4.141	155
*18*	*Journal of Parenteral & Enteral Nutrition*	SAGE Publications	4.109	86
*19*	*Obesity*	Wiley Blackwell	3.969	177
*20*	*Nutritional Neuroscience*	Taylor & Francis	3.950	51
*21*	*Food Reviews International*^*c*^	Taylor & Francis	3.933	62
*22*	*Hepatobiliary Surgery & Nutrition*	AME	3.911	–
*23*	*Journal of the International Society of Sports Nutrition*	BioMed Central	3.841	37
*24*	*Food Policy*	Elsevier	3.788	85
*25*	*Nutrition & Metabolism*	BioMed Central	3.599	68
*26*	*Nutrition Journal*	BioMed Central	3.592	70
*27*	*Nutrition*	Elsevier	3.591	128
*28*	*Current Opinion in Clinical Nutrition & Metabolic Care*^*c*^	Lippincott Williams & Wilkins	3.570	98
*29*	*International Journal of Eating Disorders*	Wiley	3.523	126
*30*	*Appetite*	Elsevier	3.501	120
*31*	*Applied Physiology Nutrition & Metabolism*	National Research Council Canada	3.455	78
*32*	*Current Obesity Reports*^*c*^	Springer	3.454	20
*33*	*Nutrition Metabolism & Cardiovascular Diseases*	Elsevier	3.340	84
*34*	*British Journal of Nutrition*	Cambridge University Press	3.319	166
*35*	*Maternal & Child Nutrition*	Wiley	3.305	51
*36*	*Journal of Functional Foods*	Elsevier	3.197	63
*37*	*European Journal of Clinical Nutrition*	Springer Nature	3.114	141
*38*	*Nutrition & Diabetes*	Springer Nature	3.098	28
*39*	*Journal of Human Nutrition & Dietetics*	Wiley	3.088	58
*40*	*Annals of Nutrition & Metabolism*	S. Karger AG	3.051	71
*41*	*Journal of Pediatric Gastroenterology & Nutrition*	Wolters Kluwer	3.015	121
*42*	*Beneficial Microbes*	Wageningen	2.939	31
*43*	*Genes & Nutrition*	BioMed Central	2.883	41

Notes:

^a^ Journals 1–21 are in JCR Quartile 1 and journals 22–43 in Quartile 2.

^b^ 42 of 43 journals have a Scimago Impact Factor score.

^c^ Journal publishes only review articles.

Table 2 shows which of the five publication procedures was mentioned in the instructions for authors of each of the ten journals that exclusively publish review articles. Conflict of interest disclosure was required by all journals, but two of the other four procedures were only mentioned by three or four journals and two were not mentioned by any journal in its instructions for authors.

**Table 2:** Specification in Instructions for Authors of Five Publication Procedures Designed to Improve Research Integrity by 2018 JCR Q1 and Q2 Nutrition and Dietetics Journals that Publish Reviews (*n* = 10)^a^

Journal^**b**^	COI Disclosure	Recommends Guidelines^**c**^	Prospero Registration	Data Sharing	Registered Reports	Total
*1 Progress in Lipid Research*	✓	✗	✗	✓	✗	2
*2 Annual Review of Nutrition*	✓	✗	✗	✗	✗	1
*3 Advances in Nutrition*	✓	✓	✗	✗	✗	2
*4 Critical Reviews in Food Science & Nutrition*	✓	✗	✗	✓	✗	2
*5 Nutrition Reviews*	✓	✓	✗	✗	✗	2
*6 Nutrition Research Reviews*	✓	✗	✗	✗	✗	1
*7 Proceedings of the Nutrition Society*	✓	✓	✗	✗	✗	2
*8 Food Reviews International*	✓	✗	✗	✓	✗	2
*9 Current Opinion in Clinical Nutrition & Metabolic Care*	✓	✗	✗	✗	✗	1
*10 Current Obesity Reports*	✓	✗	✗	✓	✗	2
Total	10	3	0	4^d^	0	17

Notes

^a^ ✓ = journal recommends use of the procedure; ✗ = journal does not recommend use of the procedure.

^b^ Journals 1 to 8 are classified as JCR Q1 and journals 9 and 10 as Q2.

^c^ PRISMA and/or MOOSE Guidelines.

^d^ The data sharing policy of the journal or publisher encourages this practice; none of the 4 require data sharing as a condition of publication.

Table 3 shows which of the six publication procedures was mentioned in the instructions for authors of the 33 nutrition and dietetics journal that published articles reporting original research. The range was 1 to 5, and the mean was 3.7. Twenty-three (70%) of the journals discussed four or more of the procedures in their instructions for authors.

**Table 3:** Specification in Instructions for Authors of Six Publication Procedures Designed to Improve Research Integrity by 2018 JCR Q1 and Q2 Nutrition and Dietetics Journals that Publish Original Research (*n* = 33)^a^

Journal	COI Disclosure	Recommends Guidelines	Clinical Trial Registration^**b**^	Other Registration	Data Sharing	Registered Reports	Total
*1 American Journal of Clinical Nutrition*	✓	✓	✓	✓	✓	✗	5
*2 Clinical Nutrition*	✓	✓	✓	✗	✓	✗	4
*3 International Journal of Behavioral Nutrition & Physical Activity*	✓	✓	✓	✓	✓	✗	5
*4 Food Chemistry*	✓	✓^c^	✓^c^	✗	✓	✗	4
*5 International Journal of Obesity*	✓	✓	✓	✗	✓	✗	4
*6 Journal of Nutritional Biochemistry*	✓	✓^d^	✓^c^	✗	✓	✗	4
*7 European Journal of Nutrition*	✓	✗	✗	✗	✓	✗	2
*8 Journal of Nutrition*	✓	✓	✓	✓	✗	✗	4
*9 Nutrients*	✓	✓^e^	✓	✗	✓	✗	4
*10 Journal of the Academy of Nutrition & Dietetics*	✓	✓	✓	✗	✓	✗	4
*11 Journal of Parenteral & Enteral Nutrition*	✓	✗	✗	✗	✗	✗	1
*12 Obesity*	✓	✓	✓	✗	✓	✗	4
*13 Nutritional Neuroscience*	✓	✓	✓	✗	✓	✗	4
*14 Hepatobiliary Surgery & Nutrition*	✓	✓	✓	✗	✗	✗	3
*15 Journal of the International Society of Sports Nutrition*	✓	✓	✓	✓	✓	✗	5
*16 Food Policy*	✓	✓	✗	✗	✓	✗	3
*17 Nutrition & Metabolism*	✓	✓	✓	✓	✓	✗	5
*18 Nutrition Journal*	✓	✓	✓	✓	✓	✗	5
*19 Nutrition*	✓	✓^f^	✓	✗	✓	✗	4
*20 International Journal of Eating Disorders*	✓	✓	✓	✗	✓	✗	4
*21 Appetite*	✓	✓^g^	✗	✗	✓	✗	3
*22 Applied Physiology Nutrition & Metabolism*	✓	✓^c^	✓ ^c^	✗	✓	✗	4
*23 Nutrition Metabolism & Cardiovascular Diseases*	✓	✓	✓	✗	✓	✗	4
*24 British Journal of Nutrition*	✓	✓	✓	✗	✗	✗	3
*25 Maternal & Child Nutrition*	✓	✓	✓	✗	✓	✗	4
*26 Journal of Functional Foods*	✓	✓^g^	✗	✗	✓	✗	3
*27 European Journal of Clinical Nutrition*	✓	✓	✓	✗	✓	✗	4
*28 Nutrition & Diabetes*	✓	✓	✓	✗	✓	✗	4
*29 Journal of Human Nutrition & Dietetics*	✓	✓	✓	✓	✗	✗	4
*30 Annals of Nutrition & Metabolism*	✓	✓^h^	✓^c^	✗	✗	✗	3
*31 Journal of Pediatric Gastroenterology & Nutrition*	✓	✓	✓	✗	✗	✗	3
*32 Beneficial Microbes*	✓	✗	✗	✗	✗	✗	1
*33 Genes & Nutrition*	✓	✓	✓	✓	✓	✗	5
TOTAL	33	30	27	8	25^i^	0	123

Notes

^a^ ✓ = journal recommends use of the procedure; ✗ = journal does not recommend use of the procedure. Rows 1–13 are JCR Q1 journals and rows 14–33 are Q2 journals.

^b^ Three journals (*Food Policy*, *Appetite* and *Journal of Functional Foods*) contained a link to their publisher which stated that it supported clinical trial registration for relevant journals. These were rated ✗ (No) in the table as the language in linked document is vague and does not state which journals are relevant.

^c^ Procedure mentioned only in a document linked to the instructions for authors, but not in the actual instructions.

^d^ One set of guidelines mentioned in the instructions for authors (ARRIVE) and one (CONSORT) mentioned only in a document inked to these.

^e^ Three sets of guidelines are mentioned in the instructions for authors (PRISMA; CONSORT; ARRIVE), and these and one other (TOP) are mentioned in a document linked to instructions.

^f^ One set of guidelines mentioned in the instructions for authors (CONSORT) and two (CONSORT; ARRIVE) mentioned in a document linked to these.

^g^ One set of guidelines mentioned in the instructions for authors (ARRIVE) and two (CONSORT; ARRIVE) mentioned in a document linked these.

^h^ Two sets of guidelines mentioned in the instructions for authors (the EQUATOR Network and PRISMA) and one (ARRIVE) mentioned in a document linked to these.

^i^ 22 of the 25 journals encourage data sharing for all types of studies. Additionally, *Obesity* and *Applied Physiology Nutrition and Metabolism* each requires a written data sharing statement for clinical trials, but not for papers reporting other study designs. Only *Nutrition and Diabetes* requires data sharing as a condition of publication.

All of the 33 journals required authors to disclose conflicts of interest. The second most widely recommended publication procedure was use of guidelines when writing-up results from empirical studies, with 30 of the 33 journals mentioning at least one specific set of guidelines and one mentioning the EQUATOR Network in their instructions to authors or related documents. Registration of clinical trials was also common, with 27 journals requiring this of investigators. Compared to clinical trials, registration of research using other study designs was much less common, with just eight journals requiring this of investigators. Of these, seven required registration of systematic reviews in PROSPERO and one (*Journal of Nutrition*) required registration of observational studies, but did not specify a particular registry.

Discussion of data sharing appeared in the instructions for authors of 25 of the 33 journals. For the vast majority, this involved encouraging data sharing for all types of studies. The three exceptions to this were *Nutrition and Diabetes* for which data sharing was a condition of publication, and *Obesity* and *Applied Physiology Nutrition and Metabolism* which both required a data sharing statement, but only for clinical trials. None of the journals had adopted the Registered Reports publication format at the time the review was conducted.

Table 4 shows the specific guidelines and guideline repositories recommended in each of the 30 journals that discussed these in their instructions for authors. Ten journals mentioned the EQUATOR Network repository of guidelines. Five of these journals also mentioned the FAIRsharing repository and all but one also mentioned a specific set of guidelines. A total of 19 specific guidelines were recommended, with the number mentioned by each journal that discussed these ranging from one to 12. Of the 29 that recommended a specific set of guidelines, 27 mentioned CONSORT, 21 ARRIVE, 19 PRISMA and 12 STROBE. Each of the other 15 guidelines was mentioned by fewer than 10 journals.

**Table 4:** Mention by Journals of the EQUATOR Network and Specific Guidelines (*n* = 30)^a^

		Specific Guidelines																			
Journal	E Q U A T O R^**b**^	A G R E E	A R R I V E	C A R E	C H E E R S	C O N S O R T	C O R E Q	E N T R E Q	M O O S E	P R I S M A	P R I S M A -P	R A T S	S A M P L	S P I R I T	S Q U I R E	S R Q R	S T A R D	S T R O B E	T O P	T R I P O D	T o t a l^**c**^
*American Journal of Clinical Nutrition*	✓		✓			✓				✓								✓			4
*Clinical Nutrition*			✓			✓				✓											3
*IJBNPA*	✓		✓	✓	✓	✓	✓			✓	✓			✓			✓	✓		✓	11
*Food Chemistry*						✓															1
*International Journal of Obesity*			✓			✓															2
*Journal of Nutritional Biochemistry*			✓																		1
*Journal of Nutrition*			✓			✓				✓											3
*Nutients*			✓			✓				✓									✓		4
*Journal of the Academy of Nutrition & Dietetics*				✓	✓	✓	✓	✓	✓	✓		✓						✓			9
*Obesity*						✓			✓	✓											3
*Nutritional Neuroscience*						✓															1
*Hepatobiliary Surgery & Nutrition*						✓															1
*JISSN*	✓		✓	✓	✓	✓	✓			✓	✓		✓	✓			✓	✓		✓	12
*Food Policy*			✓			✓															2
*Nutrition & Metabolism*	✓		✓	✓	✓	✓	✓			✓	✓		✓	✓			✓	✓		✓	12
*Nutrition Journal*	✓		✓	✓	✓	✓	✓			✓	✓		✓	✓			✓	✓		✓	12
*Nutrition*			✓			✓															2
*International Journal of Eating Disorders*	✓		✓	✓	✓	✓	✓			✓	✓			✓			✓	✓		✓	11
*Appetite*			✓			✓															2
*Applied Physiology Nutrition & Metabolism*	✓																				0
*Nutrition Metabolism & Cardiovascular Diseases*			✓			✓			✓	✓											4
*British Journal of Nutrition*			✓			✓			✓	✓											4
*Maternal & Child Nutrition*	✓	✓	✓	✓	✓	✓				✓				✓	✓	✓	✓	✓			11
*Journal of Functional Food*			✓			✓															2
*European Journal of Clinical Nutrition*						✓				✓											2
*Nutrition & Diabetes*			✓			✓															2
*Journal of Human Nutrition & Dietetics*						✓				✓								✓			3
*Annals of Nutrition & Metabolism*	✓		✓							✓								✓			3
*JPGN*						✓			✓	✓					✓		✓	✓			6
*Genes & Nutrition*	✓		✓	✓	✓	✓	✓			✓	✓		✓	✓			✓	✓		✓	12
Total^c^		1	21	8	8	27	7	1	5	19	6	1	4	7	2	1	8	12	1	6	145

Notes

^a^ *IJBNPA* International Journal of Behavioral Nutrition & Physical Activity, *JISSN* Journal of the International Society of Sports Nutrition, *JPGN* Journal of Pediatric Gastroenterology & Nutrition*.* The first 11 journals are JCR Q1 journals and the remaining 19 are Q2 journals.

^b^ Five of the 10 journals that discussed the EQUATOR Network in their instructions to authors also discussed the FAIRsharing repository: *International Journal of Behavioral Nutrition & Physical Activity*; *Journal of the International Society of Sports Nutrition*; *Nutrition & Metabolism*; *Nutrition Journal*; *Genes & Nutrition*.

^c^ The row and column totals are for specific guidelines and exclude the EQUATOR Network.

## Discussion

The requiring of Registered Reports has not been adopted at all within the discipline’s high impact academic peer-reviewed outlets, and registration of studies other than clinical trials is seldom required. Notably however, and consistent with similar studies conducted in other academic disciplines, publication procedures such as conflict of interest disclosure, clinical trial registration, recommendation of guidelines and data sharing have become standard editorial requirements in nutrition and dietetic journals.

Nevertheless, given this generally encouraging picture, the question arises as to why there is such concern about the quality and integrity of published research within the field of nutrition and dietetics studies [3–6]. In answer, the limited effects on research quality and transparency of the publication procedures examined in this study can best be explained by the mechanisms through which they influence analytic flexibility and selective reporting and the extent to which they are properly implemented and enforced. For example, conflict of interest disclosure does not directly affect flexible data analysis and selective reporting of results. Rather, it alerts readers to the fact that one or more authors of a paper has a competing interest that may create a preference for results of a certain kind (e.g., those showing a positive effect of an intervention program). In addition, journal conflict of interest disclosure policies almost exclusively focus on financial conflicts, which is a potential limitation, especially in applied research fields where affiliation and confirmation biases may predispose investigators to favor certain types of results over others. In light of such influences, it has been argued that there is a need to focus on conflicts of interest beyond those of a financial nature, such as career-related advancement and ideological conflicts [3, 36]. The scope of these may be fairly wide in the field of nutrition and dietetics research. For example, a number of the papers retracted by the Cornell University Food and Brand Lab focused on meal sizes and the structure of eating habits, ideas for which its director was well-known and about which he had written a popular book [37]. Kroger and colleagues [3] propose that investigators who have published a book relevant to the topic of their research should report this as a financial conflict of interest.

However, even in the absence of such a book, it is likely that investigators will have a strong affiliation to the theories they have developed and their prior discoveries regarding these, and therefore their ability to objectively conduct replication studies may be limited [38]. How such affiliation bias should be covered by conflict of interest disclosures remains unclear, and it is questionable whether this is even feasible and desirable [39]. Accordingly, conflict of interest disclosures may not be the most efficient editorial procedure to employ when attempting to reduce analytic flexibly and selective outcome reporting. In the absence of being able to create a list of every possible “interest” that might influence how studies are conceptualized and conducted, it is probably easier to put in place publication procedures such as prospective registration and Registered Reports that make such practices difficult to engage in, whatever the motivation for their use.

Indeed, study registration has been presented by its advocates as a more direct way to address these problems [40] and, as was found in the present study, mandatory registration is becoming widespread among academic journals, at least in the case of randomized clinical trials. However, its application beyond this type of study has been minimal [21, 23], and this was found to be the case in the current analysis of nutrition and dietetics journals. The two main limitations of study registration are that a large proportion of registered studies are retrospectively registered and many investigators do not adhere to key elements of their registered analysis plan when analyzing data and writing-up results for publication [41–44]. So, while it is encouraging that more than 80% of journals required clinical trial registration and we would recommend all journals require registration of studies using other research designs (e.g., systematic reviews, observational studies), it is essential that editors insist registration is prospective and not retrospective and that they require investigators to demonstrate they adhered to their prospectively registered analysis plans [15, 45].

Lack of adherence and suboptimal oversight is also a problem with study guidelines and data sharing, both of which tend to be recommended or encouraged, rather than required, by journals. As shown in the current study, there now exist a lot of reporting guidelines, but it is unlikely reviewers check manuscript against all the items appearing in these when reviewing manuscripts, and research shows adherence to many of the specific items described in CONSORT, PRISMA and STROBE remains poor [46–48]. If journals are to recommend use of reporting guidelines, they should ensure that there is some formal procedure used to assess adherence built into the peer-review process, with either authors or reviewers completing a check-list of compliance.

While three-quarters of the nutrition and dietetics journals in the current study discussed data sharing in their instructions for authors, only one required it as a condition of publication. We recommend all journals require a clear data sharing statement from authors giving precise reasons for their decisions as to how they are going to share their data, and justifying any decision not to share data. If data are housed in a third-party repository, the means of accessing these should be specified; if they are not stored in such a repository, a clear procedure for obtaining the data from the authors should be described. The type of voluntary policies favored by nutrition and dietetics journals have been shown to result in less than one in ten investigators making raw empirical data accessible [49–52]. Moreover, even when data are shared by investigators, someone has to reanalyze them and identify discrepancies and irregularities in order for analytic flexibility and selective reporting to be identified. For this to have a deterrent effect, a great deal of such reanalysis of published studies would need to take place, something which is unlikely in the prevailing research culture that places little value on replication studies [53]. Also, in the absence of a prespecified analysis plan, there is no way to recreate the study as initially designed and identify deviations from this in a replication study. Reproducibility also requires adherence to a prespecified analysis plan that describes the computational procedures used in the original research, as well as access to the original data and code [54].

### Limitations

There are a number of limitations of this study that should be noted. First, not all nutrition and dietetics journals were included and it is possible that other journals, such as those included in Quartiles 3 and 4 of the JCR, used more or less of any one of the particular publication procedures that were examined. Second, the instructions for authors and related materials were reviewed by just two individuals who may each have failed to identify a publication procedure in the course of their review. Third, the paper presents a snap-shot of the state of the discipline, and journals may have subsequently adopted one or more of the publication procedures assessed since the time the review was conducted. For example, we are aware that one of the journals included in our sample (the *International Journal of Eating Disorders*) has subsequently introduced Registered Reports as an option for authors. Fourth, the study presents no data as to whether the procedures examined were actually enforced by the journals that adopted them and adhered to by the authors that publish in these journals. As noted above, adherence and enforcement are essential to the effective operation of editorial policies in improving research transparency and quality.

## Conclusions

In conclusion, publication procedures that lock investigators into prespecified hypotheses, methods, measures and statistical analyses have the greatest potential to limit analytic flexibility and selective reporting of results. Of the procedures examined in this study that best accomplish this, one (Registered Reports) was not used by any of the nutrition and dietetics journals reviewed and the other (registration) was rarely required outside of clinical trials. None of the journals that published only reviews and meta-analyses required that these be preregistered.

We recommend that all nutrition and dietetics journals adopt one or both of the procedures that lock-in data analyses and allow differentiation of genuine confirmatory hypothesis testing from other types of research. Journals could then clearly distinguish (either by having separate sections or including some indicator on the title page of each paper) confirmatory research from research that purports to be but cannot demonstrate that the hypotheses tested were pre-specified. A third category of genuine exploratory research could also be included. The journal instructions for authors could clearly specify the types of claims that can be made for studies within each of these categories. This simple procedure would prevent investigators who employ flexible data analysis practices and selective reporting from presenting the results of these as genuine hypothesis testing [13]. The goal would be, over time, to move more investigators to preregister their hypotheses and analysis plans or publish using the registered reports format in order that their papers be published in the more prestigious confirmatory category that allowed stronger statements about the validity and implications of the study. At present, nutrition and dietetics journals make no distinction between results that emerge from flexible data analysis and selective reporting and those that do not, and therefore there is no incentive for investigators to prespecify their hypotheses and analysis plans.

## Data Availability

The dataset generated and analyzed during the current study are available from the corresponding author on reasonable request.

## Abbreviations

ARRIVE: Animal Research: Reporting of in Vivo Experiments
CONSORT: Consolidated Standards for Reporting Trials
EQUATOR: Enhancing the QUAlity and Transparency Of health Research
JCR: Journal Citation Report
MOOSE: Meta-analysis Of Observational Studies in Epidemiology
PDF: Portable Document Format
PRIMSA: Preferred Reporting Items for Systematic Reviews and Meta-Analyses
SJR: Scimago Journal Ranking
STROBE: Strengthening the Reporting of Observational Studies in Epidemiology
